# Editorial: Nutritional Aspects of Kidney Disease

**DOI:** 10.3389/fnut.2021.803351

**Published:** 2021-11-17

**Authors:** Alice Sabatino, Carla Maria Avesani

**Affiliations:** ^1^Dipartimento di Medicina e Chirurgia, Università di Parma, Parma, Italy; ^2^Medical Unit Clinical Nutrition, Division of Renal Medicine and Baxter Novum, Department of Clinical Science, Technology and Intervention, Karolinska Institutet, Karolinska University Hospital, Stockholm, Sweden

**Keywords:** kidney disease, nutrition, chronic kidney disease, acute kidney injury, body composition, end-stage kidney disease, dialysis

The kidneys are homeostatic organs that remove waste products from the metabolism and regulate the concentration of mineral, ions and the volume of body fluids. In the presence of kidney disease (KD) this function is compromised, leading to accumulation of water, minerals and waste products in the body. A close nutritional follow-up which comprises periodical nutritional assessment and supervised dietary modification is key for the management of KD (1, 2), playing an important role in reducing the risk for cardiovascular disease and managing the clinical effects of the reduced glomerular filtration rate, while keeping an optimal nutritional status (3).

This recent Research Topic on “Nutritional aspects of kidney disease” represents a collection of 11 articles, including 8 original research articles, ranging from pathophysiological-related mechanisms to body composition assessment, dietary patterns, and clinical counseling in the field of chronic kidney disease (CKD) and acute kidney injury (AKI). A common theme across the Research Topic is the assessment of nutritional status and body composition of patients with KD. This is an important topic for investigation since protein energy wasting (PEW) and sarcopenia are common in patients with KD, and are associated with adverse clinical outcomes (4, 5). In the current issue Macedo et al. showed that older adults on hemodialysis can concomitantly have malnutrition and sarcopenia, and that both conditions can be independently present in this patient group even if overweight is present. In addition, the authors also demonstrated that malnutrition and sarcopenia together have a worse quality of life and survival.

Many are the factors involved in the worsening of nutritional status of patients with CKD, including unsupervised dietary modifications, which in conjunction with the loss of appetite often observed in this patient group, may lead to spontaneous reduction in energy and nutrient intake (6). Moreover, the catabolic effects of kidney replacement therapy (KRT), metabolic and hormonal derangements, the presence of systemic inflammation and comorbid conditions, and also reduced physical activity contribute to a state of negative energy and protein balance both in patients with AKI and patients with CKD/ESKD (6, 7). A recent hypothesis is that patients undergoing dialysis are more “anabolic resistant,” characterized by blunted stimulation of muscle protein synthesis to common anabolic stimuli such as food intake and physical activity. Garibotto et al. thoroughly discuss this hypothesis in a very interesting and in-depth review, bringing awareness to a problem that is far from being solved.

Unique aspects of KD, such as fluid overload and consequent body weight fluctuations, hinder a reliable assessment of nutritional status, and consequently, the management of these conditions. This topic gathers much interest and most papers from this special collection discuss or investigate different tools to assess body composition in patients with KD. Sabatino et al. presented the results of an observational study that investigated the role of ultrasound (US) in the monitoring of quadriceps muscle mass of patients with AKI. They observed an important reduction in quadriceps muscle mass in the first 5 days in the ICU stay, which is expected given the strong catabolic stimuli present in this clinical setting. Overall, this paper provides evidence that US is emerging as a sensitive alternative for the body composition assessment of patients with KD. Aligned with this idea, Reis et al. investigated the role of electrical bioimpedance (BIA) in patients on peritoneal dialysis, showing how single frequency BIA seems to be more accurate than multi-frequency BIA in this clinical setting. Finally, Bellafronte et al. showed that predictive equations applied to estimate skeletal muscle mass from BIA parameters can be useful for the identification of patients with low muscle mass. In addition, Sabatino et al. discuss in a narrative review the usefulness of different bedside tools currently studied and/ or applied in the clinical practice for patients on hemodialysis. An emphasis is given to simplified creatinine index [SCI], bioimpedance spectroscopy (BIS) and muscle US, all suitable for clinical practice use. From these, US is the only method that measures muscle in different dimensions, while BIS and SCI are dependent on either theoretical assumptions or on the use of population specific regression equations, which may lead to errors when fluid alteration (over or dehydration) is present, a condition often observed in patients with KD.

A decline in physical function is also of much concern in this clinical setting, being related with increased mortality, falls, fractures, disability and low quality of life (8). Although malnutrition, sarcopenia and low physical function partially overlap, they do not develop in the same rate nor necessarily at the same time (4). In this regard, Mota Silva et al. demonstrated how low lean mass as assessed by phase angle from BIA, but not inflammation and overhydration, has a moderate but statistically significant correlation with worse physical function in patients on peritoneal dialysis.

Another important challenge in the current nutritional management of patients with end stage kidney disease (ESKD) on hemodialysis is the achievement of a good dietary quality pattern (9). The fear of hyperkalemia often precludes clinicians from recommending patients to eat fruits and vegetables, which results in poor fiber intake. A low-fiber dietary intake seems to associate with the development of low grade systemic inflammation through the promotion of intestinal dysbiosis and should not be overlooked (10). Other unwanted consequences, especially in this clinical setting, may arise from a pro-inflammatory dietary habit. Qin et al. show how pro-inflammatory dietary patterns, as assessed by the dietary inflammatory index (DII), positively correlated with parathyroid hormone (PTH) and increased the risk of hyperparathyroidism in patients with early stages of CKD. Although it is a challenge to improve diet quality; tabus, pre-conceptions and non-evidence based dietary counseling regarding the role of fruits and vegetables in the development of hyperkalemia must be resolved, and dietitians should be called into action. That is the theme of a very interesting article published in the current Research Topic. Chan et al. evaluates the impact of individualized nutritional counseling in improving diet quality in patients with CKD, showing encouraging results.

Finally, complementing this special collection, Wang et al. describe the risk factors for renal impairment in adult patients with short bowel syndrome; and Graidis et al. discuss the role of vitamin D in the development and prognosis of AKI as well as a therapeutic adjuvant.

To summarize, we understand that this volume brings a conjunction of papers that makes up a translational point of view for the medical nutritional therapy of kidney diseases—all aligned with the unanswered questions pointed as topics requiring investigation by the KDOQI 2020 Clinical Practice Guideline for Nutrition in Chronic Kidney Disease (1). We are certain that this reading will add new information for clinicians and researchers in the field of nutrition and kidney diseases.

## Author Contributions

All authors listed have made a substantial, direct and intellectual contribution to the work, and approved it for publication.

## Conflict of Interest

The authors declare that the research was conducted in the absence of any commercial or financial relationships that could be construed as a potential conflict of interest.

## Publisher's Note

All claims expressed in this article are solely those of the authors and do not necessarily represent those of their affiliated organizations, or those of the publisher, the editors and the reviewers. Any product that may be evaluated in this article, or claim that may be made by its manufacturer, is not guaranteed or endorsed by the publisher.
